# Acquired heterotopic ossification in hips and knees following encephalitis: case report and literature review

**DOI:** 10.1186/1471-2482-14-74

**Published:** 2014-10-03

**Authors:** Xianghong Zhang, Shuo Jie, Tang Liu, Xiangsheng Zhang

**Affiliations:** 1 Department of Orthopedics, the Second Xiangya Hospital, Central South University, 139 Renmin Road, Changsha, Hunan 410011, P.R. China

**Keywords:** Heterotopic ossification, Encephalitis, Hip, Knee

## Abstract

**Background:**

Heterotopic ossification (HO) is a rare and potentially detrimental complication of soft-tissue trauma, amputations, central nervous system injury (traumatic brain injuries, spinal cord lesions, tumors, encephalitis), vasculopathies, arthroplasties and burn injury, characterized by lamellar bone growth in non-osseous tissues such as the muscle and the joint capsule. Heterotopic ossification associated with encephalitis is rare and the occurrence of excessive, symptomatic heterotopic ossification around bilateral hips and bilateral knees is rarely described in the literature.

**Case presentation:**

We present a 47-year-old man with heterotopic ossification in the bilateral hips and bilateral knees that prevented him from walking after being attacked by encephalitis as the case study. He developed severe pain and significantly impaired range of motion of bilateral hips and bilateral knees. Research so far revealed that the management of heterotopic ossification is controversial. After requiring revision surgery resection of heterotopic ossification, reconstruction of the medial collateral ligament and adjunctive pharmacotherapy of 200 mg Celecoxib for 8 weeks after operation, he regained mobility of his joints. On review of X-ray, there was no recurrence of HO and no loosening of rivets which were used in the reconstruction of medial collateral ligament.

**Conclusion:**

Heterotopic ossification in the bilateral hip joints and bilateral knee joints associated with encephalitis have never been reported previously. Daily functions of heterotopic ossification patients can be hampered by pain, inflammation, reduced mobility, the loss of normal posture and other complications. Further studies of presumptive root causes, the early diagnosis, preventability and optimal therapeutic measures for heterotopic ossification following encephalitis are required. Different patient should be managed with different appropriated protocol based on the risk of individual patient and the institutional experience.

## Background

Heterotopic ossification (HO) is a pathological process of lamellar bone formation in soft tissue outside of skeleton. HO occurs frequently after severe head injury, spinal injury, non-traumatic intracranial lesion and long-term coma [1,2]. However, the occurrence of excessive, symptomatic heterotopic ossification around bilateral hips and bilateral knees is rarely described in the literature. Daily functions of HO patients can be hampered by the loss of normal posture, pain, inflammation, reduced mobility, formation of pressure ulcers, deep venous thrombosis and other complications [3]. Also there is a limited number of cases in the medical literature where the condition affects one or two anatomical regions in association with encephalitis. The etiopathogenesis of HO is unknown and there is lack of consensus on treatment modalities [4].

## Case presentation

A 47-year-old man, with no past medical history, suddenly complained of headaches, without fever, vomiting and altered consciousness on February 10, 2012. He was admitted to a nearby clinic, but it was useless and the headache got aggravated, following hyperpyrexia. To strive for better treatment, he got admitted into the neurology department of a superior hospital. After admission, there were apparent cognitive deficits, namely: disorientation, short attention span, and gradual disordered consciousness which progressed into coma. A magnetic resonance imaging (MRI) of the brain was unremarkable. Examination of cerebrospinal fluid and cerebrospinal fluid cultures were negative, so he was then treated for presumed viral encephalitis. According to the profile, results of blood test and imaging evidence, the patient was treated with antiviral therapy, antiepileptic therapy and empirical antibiotics within 30 days period of coma. After this period of 30 days under coma, his mental state and speech improved with vigorous and effective treatment, but the ambulation did not improve significantly. Therefore, he had difficulty using his lower extremities.

**Figure 1:**
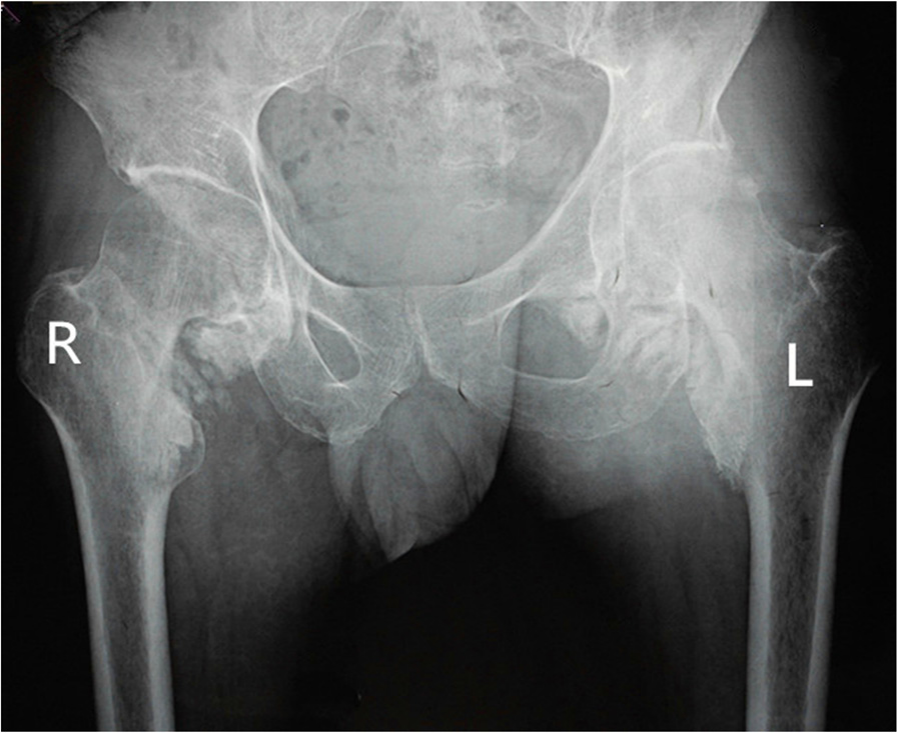
Preoperative radiographs of the pelvis shows para-articular HO on the interior aspect of both the femoral necks.

**Figure 2:**
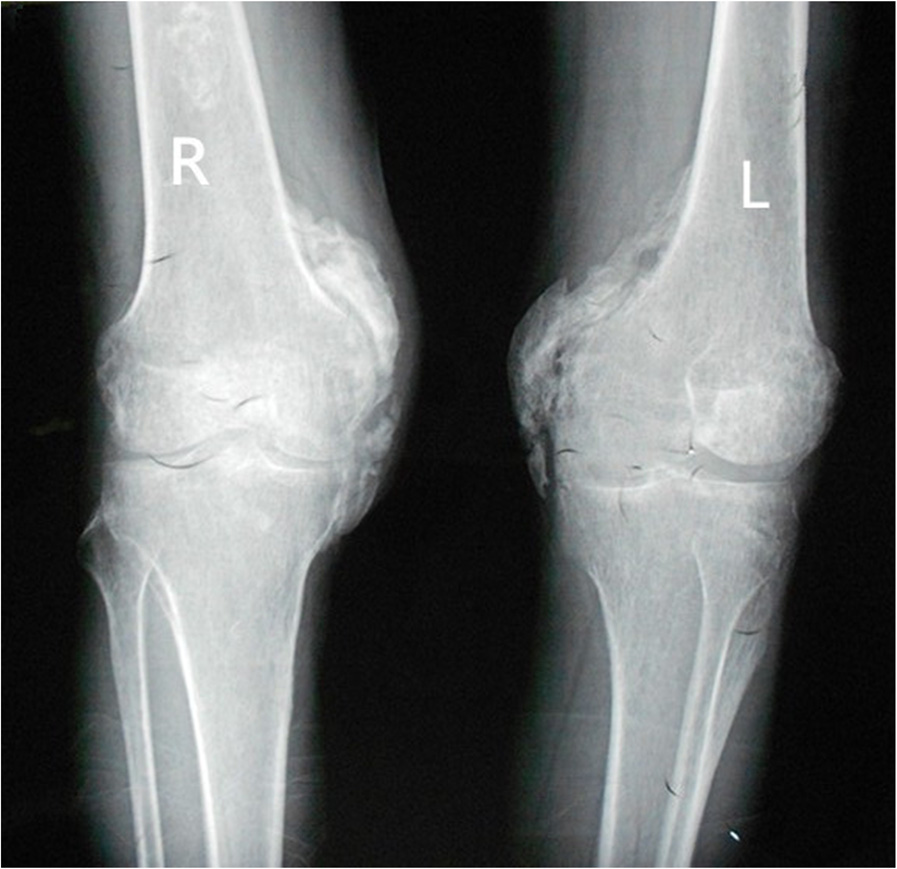
Preoperative radiographs of anterioposterior and lateral radiographs of knees shows HO on the peripheral areas of knee-joints, especially on the interior.

**Figure 3:**
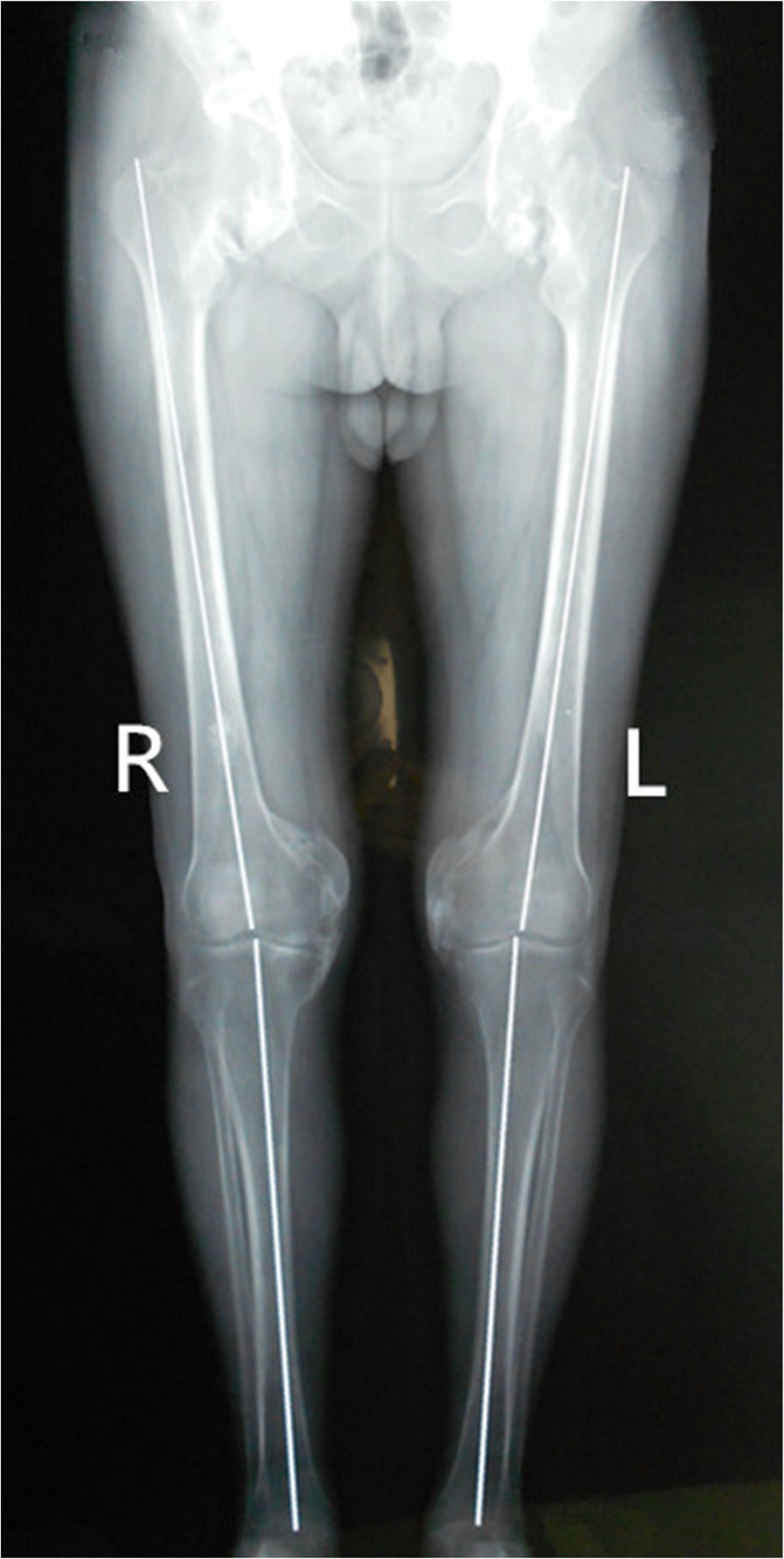
Preoperative radiographs of both lower extremities shows Lesions of four joints.

From then on, he consulted with several doctors from time to time regarding the stiffness of both the hips and knees, but the condition of his lower extremities did not improve. Ten months after the onset of the coma, he came to our department of orthopedics in order to pursue natural movement. Both hips and knees were stiff and the clinical evaluation revealed the following passive ranges of motion of hips and knees as showed in the Table 1 below. All other joints were normal.The patient’s laboratory findings were normal except for a slight increase in erythrocyte sedimentation rate (ESR) (24 mm/h; normal, 0-15 mm/h). Radiographs of the pelvis showed para-articular HO on the interior aspect of both femoral necks (Figure 1). Anterioposterior and lateral radiographs of knees (Figure 2) showed HO on the peripheral areas of knee-joints, especially on the medial. Lesions of four joints were also clearly showed in radiographs of both lower extremities (Figure 3).

**Table 1:** The comparison of range of motion between pre-operation and post-operation

	**Flexion**			**Extension**		
	**Pre-operation**	**13 months after the operation**	**18 months after the operation**	**Pre-operation**	**13 months after the operation**	**18 months after the operation**
Left hip	0°-25°	0°-85°	0°-110°	Flexion deformity	0°-15°	0°-15°
Right hip	0°-15°	0°-80°	0°-105°		0°-10°	0°-10°
Left knee	0°-15°	0°-100°	0°-125°		0°	0°
Right knee	0°-10°	0°-90°,	0°-127°		0°	0°

**Figure 4:**
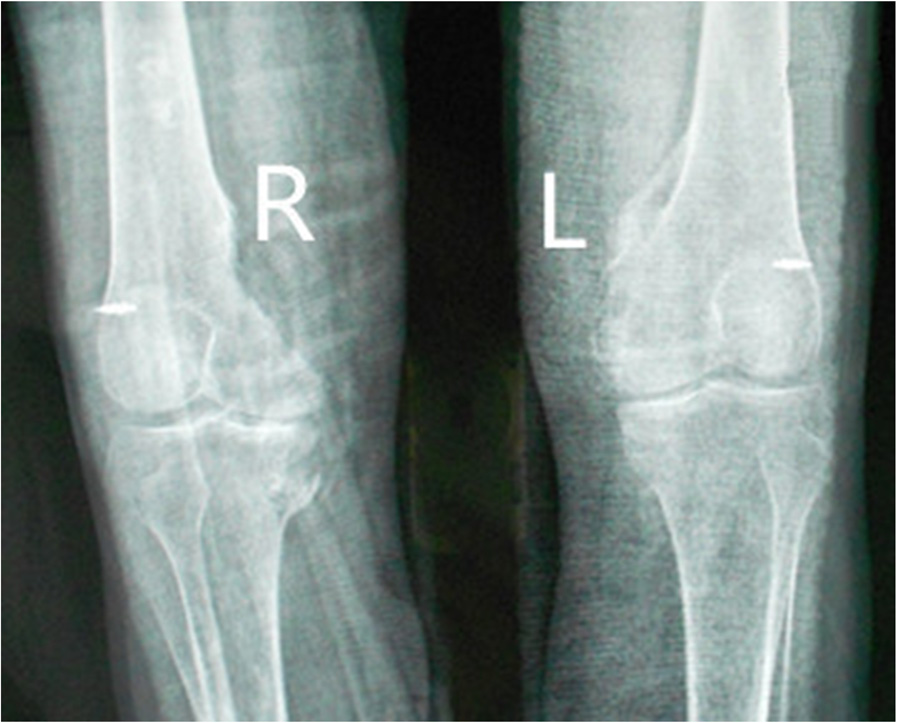
Postoperative radiographs of anterioposterior radiographs of knees shows no loosening of rivets and no recurrence 13 moths after the excision of the ossific mass.

**Figure 5:**
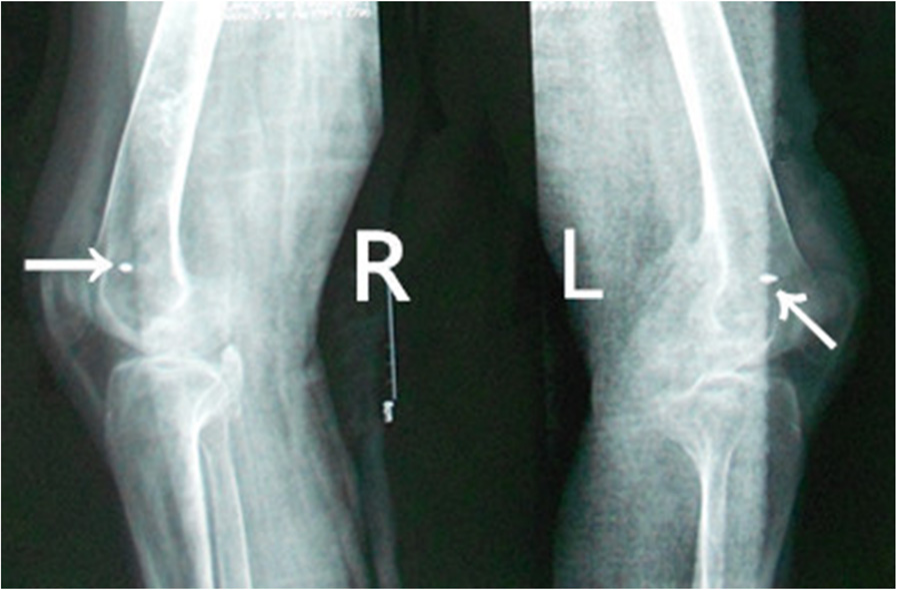
Postoperative radiographs of lateral radiographs of knees shows no loosening of rivets and no recurrence 13 moths after the excision of the ossific mass.

**Figure 6:**
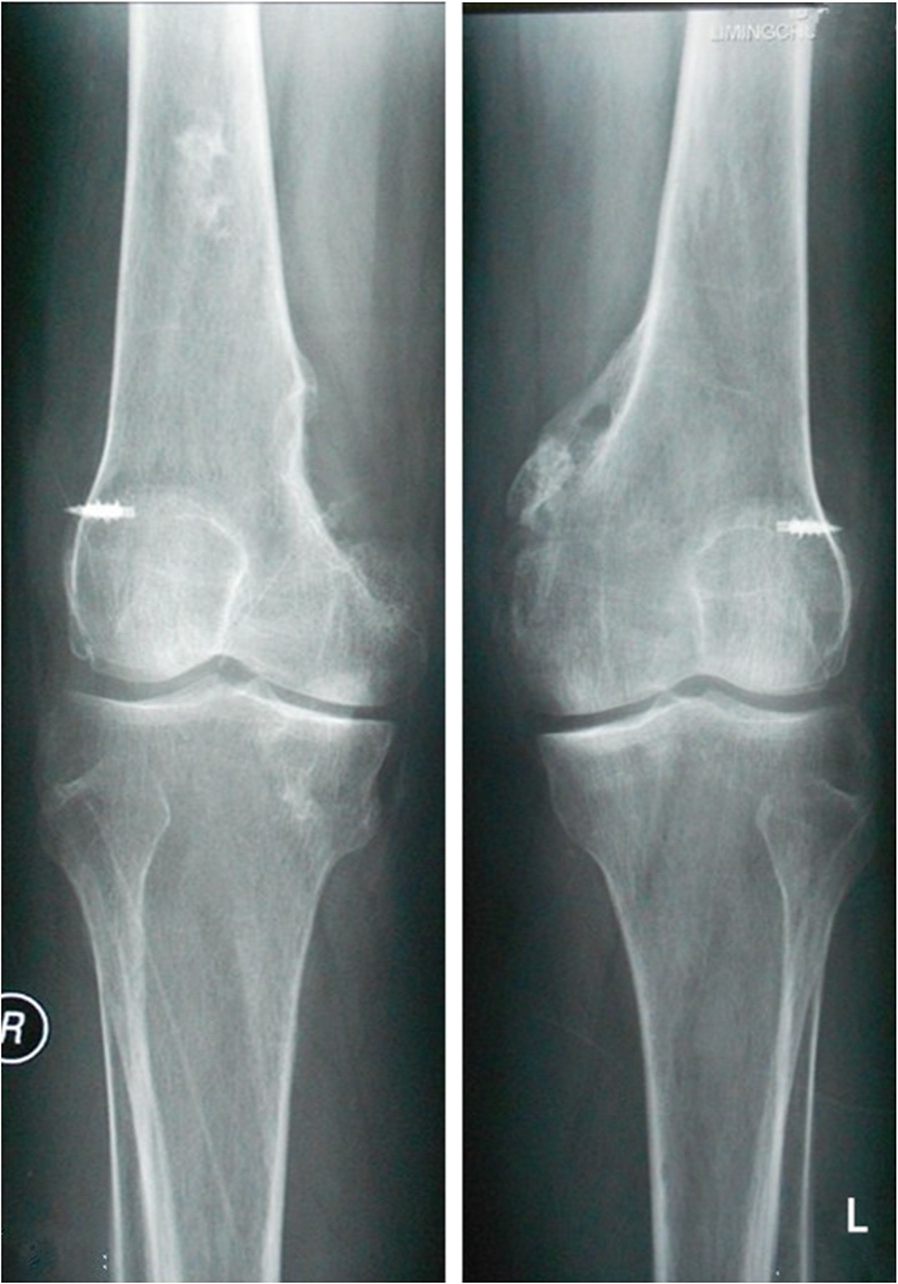
Postoperative radiographs of knees shows no loosening of rivets and no recurrence 18 moths after the excision of the ossific mass.

**Figure 7:**
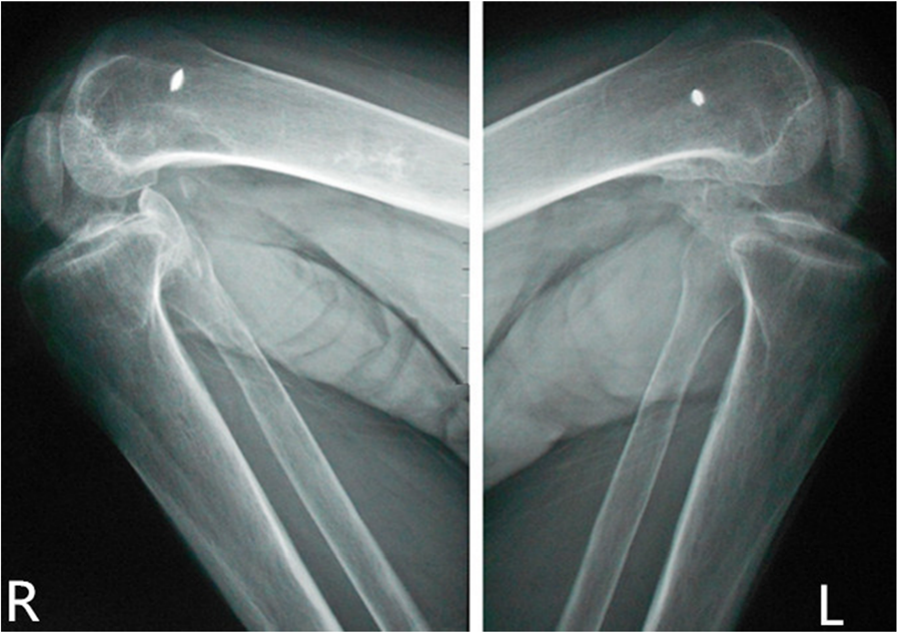
Postoperative radiographs of knees shows the passive range of motion of knees had significantly improved.

After admission to our institution, the patient and his family received pre-operation counseling, including the explanations of the available management options, expected outcome and possible complications. After extensive discussion of the risks and benefits of revision surgical excision of HO resection and concomitant peri-operative pharmacotherapy of Celecoxib for decreasing the risk of HO recurrence, the patient and his family agreed to proceed with the procedure as discussed. On December 5, 2012, he underwent the first operation: Revision surgical excision of the heterotopic bone of both knees was performed. In the surgery, an osteotome was used to excise the ossific mass in sufficient amount to free the joints and the medial collateral ligaments of both knees had degeneratively changed. Therefore, the reconstruction of the medial collateral ligament was applied. One month later, he underwent another revision surgery of excision of the heterotopic bone of both hips. The patient tolerated the surgical procedures well. After operation, the patient was given 200 mg Celecoxib orally once a day for a total of 8 weeks postoperatively. Postoperative radiographs of both knees showed that most of heterotopic bone of both knees had been excised (Figures 4 and 5). At the last follow-up visit, 18 months after the operation (is 28 months after the coma), he had no pain and could walk independently. The passive ranges of motion of hips and knees had significantly improved and the detailed information can be seen in above chart (Table 1). On review of X-ray, there was no recurrence of HO and no loosening of rivets used in the reconstruction of medial collateral ligament (Figures 6 and 7).

### Discussion

HO is a rare and potentially detrimental complication of soft-tissue trauma, amputations, central nervous system injury (traumatic brain injuries, spinal cord lesions, tumors, encephalitis) [5,6], vasculopathies, arthroplasties and burn injury, characterized by lamellar bone growth in non-osseous tissues such as the muscle and the joint capsule. After brain or spinal cord injury, trauma, neurologic disease or injuries, or hereditary disease, HO may occur [4]. HO associated with viral encephalitis is very rare [7]. The occurrence of excessive, symptomatic heterotopic ossification around bilateral hips and bilateral knees is rarely described in the literature. Our report demonstrates heterotopic bone arising from periarticular region of both hips and both knees in a patient who suffered one month prolonged coma following encephalitis. To the best of our knowledge, this is the first report of HO in both hips and both knees following encephalitis. Under reviewing literature, we made a simple chart (Table 2) below to show distinctions between patients who suffered multiple-joint following encephalitis. Daily functions of heterotopic ossification patients can be hampered by pain, inflammation, reduced mobility, the loss of normal posture and other complications [3]. Research so far reveal that the management of HO is controversial. As supported by the successful outcome in the present study, we recommend considering HO resection in conjunction with peri-operative pharmacotherapy of Celecoxib. In our current case study, the patient suffered stiffness of hips and knees and his family received pre-operation counseling, of which on December 5, 2012 he underwent the first operations. In order to free the both knee joints, We removed sufficient ossific mass and reconstructed the medial collateral ligament, This is the significant difference to other reported cases in literature which can also make the joint more stable. One month later, he underwent another revision surgical resection of the heterotopic bone of both hips. The patient tolerated the surgical procedure well. After operation, the patient was given medical treatment of 200 mg Celecoxib orally once a day for a total of 8 weeks. He had no recurrence of HO and no loose rivets for 18 months after the surgery with a significantly improved ranges of motion of hips and knees described in detail above.

**Table 2:** A brief summary about patients associated with HO following encephalitis

**Authors**	**Age/gender**	**Time of coma**	**The affected joints**	**Locations**	**Therapies**	**Consequence**
Tay [18] et al.	26/female	28 days	Bilateral hips	Image of CT showed: medial aspect of upper thighs	Surgery and radiotherapy	No recurrence and moderate gain in the rage of motion
Jayasundara [8] et al.	21/male	35 days	Bilateral hips and right elbow	CT showed: anterior,posterior and lateral aspects of both hips,	Surgery and physiotherapy	No recurrence and have acceptable range of movements at the affected joints
				Radiograph of the pelvis showed: anteroposterior		
Saito [30] et al.	26/female	40 days	Bilateral knees	CT showed: Large area of the left distal femur and small area of right knee	Surgery and 800 mg daily of etidronate disodium	Slight recurrence occurred 3 weeks after surgery
An [7] et al.	38/female	13 months	Right shoulder and left elbow	Unknown	Excision,physical and diphosphonate postoperation	No recurrence and the range of motion improved markedly
Ours case	47/male	1 month	Both hips and knees	interior aspect of both the femoral necks and the peripheral areas of knee-joints, especially interior	Excision and 200 mg daily of Celecoxib	No recurrence and walk independently

The mechanism and pathophysiology which can lead to HO formation is still not fully understood. And molecular mechanisms of HO have not been fully elucidated. Many studies showed that primitive mesenchymal cells differentiate into osteoblasts which would lead to HO formation and the origin of these mesenchymal cells and the stimulus are poorly understood [8]. Gannon FH et al. discovered that inflammation first occurs in response to stimulations, including surgery, trauma and viral illnesses [9]. Inflammatory and skeletogenic signaling pathways are also supposed to play critical roles in HO formation. Bidner SM et al. [10] proposed that failure of control in the immune system, central nervous system or indigenous inflammatory response lead to the release of inducing agents, resulting in HO formation. Urist [11] et al. discovered that demineralized bone matrix can induce the formation of HO. They also presented bone morphogenetic protein as the true inductor. Ho SSW [12] et al., recently put forward that Prostaglandin E2 is a transmitter to promote the original cell differentiation. Chalmers J [13] et al. proposed that the following three requirements are necessary for HO formation, namely: inducing agent, osteogenic precursor cell and an environment which is permissive to osteogenesis. Once these conditions are meet, mesencnchymal cells are recruited, which then proliferate and differentiate into chondrocytes and/or osteoblasts, and ultimately lead to ectopic bone formation [3]. Botolin et al. [14], also put forward that both the reaming debris and the extent of traumatic intraoperative injury to the surrounding soft tissues at the operative site play important roles in the development of HO after antegrade reamed femoral IMM in their case study. Several studies also have demonstrated that the low-oxygen tension [15] and neurotransmitters are involved in the process of HO formation. Glrland DE et al. [16] told us that prolonged coma, mechanical ventilation, spasticity and limited extremity movements may be the initiators of neurogenic HO. Although the pathogenic mechanism of HO remains unclear, functional immobility has been reported to be a risk factor [17]. Therefore, it is obvious that further studies for the mechanism and pathophysiology of HO are required.

It is also difficult to ascertain when the HO formation begins accurately, because the subject of HO formation has no specificity [18] and lacks a reliable method for early diagnosis. Atypical early clinical performance of HO are the causes of hard distinction from cellulitis, osteomyelitis, thrombophlebitis and tumor. Without early detection or intervention, progression of HO can lead to severe long-term effects, including restricted joint mobility, severe pain and nerve entrapment. In the aspect of biochemical markers, alkaline phosphatase (ALP) has some certain clinical significance of early diagnosis of HO [19], but ALP has no specificity. Therefore, it is difficult to determine the onset of HO, including HO following encephalitis in using biochemical markers. HO at different stages shows different imaging characteristics. Someone thought magnetic resonance imaging (MRI) is the best effective method for the diagnosis of early ectopic ossification, whiles X-ray and computed tomography (CT) can be used for review. Practice literature reports tell us that X-ray can not discover HO until 4–6 weeks later [20]. While some literature show that the most sensitive imaging modality for early detection of HO is three-phase bone scintigraphy which can also monitor the metabolic activity and degree of maturity of HO [19,21]. Although radiographic techniques such as computed tomography and magnetic resonance image provide high detailed anatomic representation of late stage HO, these modalities cannot detect early stages of HO. In summary, current imaging modalities, including CT, MRI and three-phase bone scintigraphy through helpful in late diagnosis are inadequate to help clinicians detect early HO development. The formation of HO begins within days to weeks of the inciting event. The disease has already spread beyond the point where it can be treated and impeded with oral medications, once visible through these current techniques. That is to say, none of the available prophylactic measures would affect the outcome of HO once the process begun [22]. Therefore, a urgent need exists to improve the current diagnostic modalities for HO which are inadequate to diagnose and intervene on HO at early time-points. Many researches showed that Roman Probe propelled non-invasive, transcutaneous evaluation of heterotopic bone formation. Petrson JR [23] et al. also suggested that Roman Spectroscopy allowed for detection of HO formation as early as 5 days in mice following a burn injury. Hence, we should try hard to develop novel screening techniques to visualize and detect the onset and progression of HO with high sensitivity and specificity.

Numerous treatment options, including pharmacotherapy (such as: non-steroidal Anti-inflammatory drugs (NSAIDs) [24], disodium etidronate (EHDP) et al.), motortherapy, radiotherapy, surgical therapy et al., are available but decision on which modality to choose depends on a detailed and accurate assessment of the disease process. NSAIDs were recognized as the most effective drugs to prevent the formation of HO after operation of acetabulum fracture [25]. Most doctors agree that indomethacin is the best choice among NSAIDs not only prevent HO but also slows down the process of HO development. However, the application of NSAIDs is relatively limited, for its adverse drug reaction such as gastrointestinal ulceration, decreased platelet aggregation and renal toxicity. Coventry MB [26] et al. conducted a research with patients who had HO following total hip arthroplasty, and they believed that radiation aids to prevent the formation of ectopic bone. However, the potential side effect that we should consider is carcinogenesis. Despite the risk that it can trigger another round of HO, surgery remains the only treatment option to date once bone tissue has formed. To increase the range of movements of the joints and improve function and quality of life, surgery was a good choice among treatments. Therefore, we choose the method of revision surgical resection in our case study.

However, it is difficulty to decide when the best time is for the revision surgical resection of HO? Many researches have indicated that there is a need to wait for the heterotopic bone to matured before the procedure is undertaken. Garland et al. suggested that it requires 18-month before surgical excision in order to allow the bone to mature [27]. And most scholars recommended a minimum wait of 1 year after ectopic bone formation ahead of surgical excision [28]. Garland DE recommended that HO resection should be performed at different time intervals according to the HO aetiology: traumatic HO should be resected at 6–9 months, spinal cord injury at 1 years and traumatic brain injury HO at 1.5 years [29]. Serum alkaline phosphatase (ALP) is an important factor we should use to determine the timing of HO resection. Though the maturity of heterotopic bone is difficult to evaluate, it is also important to prevent the high recurrence rate relevant to excision of immature ectopic bone [30]. Recurrence is also an important complication of HO which should be considered after excision. The risk of recurrence is higher in patients undergoing multiple operations sequentially [31] and it is found out that the risk of recurrence HO was high if the three or more joints were involved [27]. Botolin et al. supposed that diligent intraoperative care of the soft tissues and copious fluid irrigation with saline in the procedure of revision surgery appear to decrease the recurrence rate [14]. In order to free the stiffness in joint because of HO, it is necessary to remove sufficient ossific mass but complete excision is not necessary. And to decrease the recurrence rate of HO, active exercises should begin after the first postoperative week [27].

In summary, as major treatment options discussed above have negative side effects to some extent, it is important to evaluate the risk of individual patients, and provide safe and effective treatments for them. Further studies for the mechanism and pathophysiology, the early diagnosis and optimal managements of HO following encephalitis are required.

## Conclusion

Heterotopic ossification in the bilateral hip joints and bilateral knee joints associated with encephalitis have never been reported previously. Daily functions of heterotopic ossification patients can be hampered by pain, inflammation, reduced mobility, the loss of normal posture, and other complications. Therefore, to fully understand the pathogenesis of HO and to determine its risk factors, root causes and preventability of this potentially detrimental complications, further study is required. Different patient should be managed with a different appropriated protocol based on the risk of individual patient and the institutional experience.

## Consent

Written informed consent was obtained from the patient for publication of this Case report and any accompanying images. A copy of the written consent is available for review by the Editor of this journal.

## Competing interests

The authors declare that they have no competing interests.

## Authors’ contributions

XZ accountable for the execution of the case report, the integrity and analysis of the data and the writing of the manuscript. SJ accountable for the analysis of the data and the writing of the manuscript. TL accountable for the conception and execution of the case report. XZ accountable for the conception and execution of the case report. All authors read and approved the final manuscript.

## Authors’ information

Xianghong Zhang and Shuo Jie are co-first authors.

## Pre-publication history

The pre-publication history for this paper can be accessed here:

http://www.biomedcentral.com/1471-2482/14/74/prepub
